# MgO Nanoparticle Modified Anode for Highly Efficient SnO_2_‐Based Planar Perovskite Solar Cells

**DOI:** 10.1002/advs.201700031

**Published:** 2017-05-02

**Authors:** Junjie Ma, Guang Yang, Minchao Qin, Xiaolu Zheng, Hongwei Lei, Cong Chen, Zhiliang Chen, Yaxiong Guo, Hongwei Han, Xingzhong Zhao, Guojia Fang

**Affiliations:** ^1^ Key Lab of Artificial Micro‐ and Nano‐Structures of Ministry of Education of China School of Physics and Technology Wuhan University Wuhan 430072 P. R. China; ^2^ Wuhan National Lab on Opto‐electronics Huazhong University of Science and Technology Wuhan 430074 P. R. China

**Keywords:** hole‐blocking layers, interfaces, MgO, perovskite solar cells, surface modification, transparent conductive electrodes

## Abstract

Reducing the energy loss and retarding the carrier recombination at the interface are crucial to improve the performance of the perovskite solar cell (PSCs). However, little is known about the recombination mechanism at the interface of anode and SnO_2_ electron transfer layer (ETL). In this work, an ultrathin wide bandgap dielectric MgO nanolayer is incorporated between SnO_2_:F (FTO) electrode and SnO_2_ ETL of planar PSCs, realizing enhanced electron transporting and hole blocking properties. With the use of this electrode modifier, a power conversion efficiency of 18.23% is demonstrated, an 11% increment compared with that without MgO modifier. These improvements are attributed to the better properties of MgO‐modified FTO/SnO_2_ as compared to FTO/SnO_2_, such as smoother surface, less FTO surface defects due to MgO passivation, and suppressed electron–hole recombinations. Also, MgO nanolayer with lower valance band minimum level played a better role in hole blocking. When FTO is replaced with Sn‐doped In_2_O_3_ (ITO), a higher power conversion efficiency of 18.82% is demonstrated. As a result, the device with the MgO hole‐blocking layer exhibits a remarkable improvement of all *J–V* parameters. This work presents a new direction to improve the performance of the PSCs based on SnO_2_ ETL by transparent conductive electrode surface modification.

## 1 Introduction

Perovskite solar cells (PSCs) have attracted considerable research interest because of their excellent photovoltaic performance and simple fabrication process.^1^, ^2^ Perovskite materials have many advantages such as excellent charge‐carrier mobility, effective ambipolar charge transfer, and high optical absorption coefficient.[[qv: 1g,3]] Perovskite materials can be coated on the compact electron transport layer (ETL) immediately to form a planar heterojunction structure.[[qv: 3b,f,4]] Many semiconductors, such as TiO_2_ and ZnO, are proved to be good ETLs.^5^ But for the TiO_2_ ETL, the electron recombination rates are very high due to the low electron mobility and the ZnO ETL suffers from the issue of chemical instability. Recently, tin oxide (SnO_2_) has emerged as a promising candidate of ETL, which shows much higher electron mobility, good antireflection, low‐temperature process, and no ultraviolet (UV) photocatalysis effect in PSCs.^6^ Further efforts are made to improve the performance of PSCs with planar structure based on SnO_2_ ETLs.

It is noted that a compact ETL is a basic and essential component in PSCs for blocking holes and transporting electrons.^7^ For the ETLs, it is necessary to form a layer thick enough to extract electrons and block holes efficiently, while the thick ETLs may suffer from poor transmittance and high series resistance, which are detrimental to the performance of PSCs. However, a thin ETL might not be capable of passivating the defects efficiently and the current leakage is inevitable. Any pinholes in the compact layer can lead to shunt pathways and direct contacts between CH_3_NH_3_PbI_3_ active layer and transparent conductive oxide substrate, such as SnO_2_:F(FTO) or Sn‐doped In_2_O_3_ (ITO), resulting in high leakage current and serious charge carrier recombination at the interface.^8^ It has been reported that the losses of photoinjected electrons in PSCs correspond to the electron–hole recombination in the carrier transport process from perovskite to electrode^9^ and the trap state recombination within compact electron transport layer itself.^10^ Therefore, due to these limitations, single electron transport layer cannot suppress the carrier recombination effectively and may not be an optimal blocking layer for PSCs.^11^

Some researchers have made attempts to incorporate a new layer into ETL to form a bilayer, which were demonstrated to be effective ways of improving the interfacial behavior and the photovoltaic performance of PSCs.^12^ Chen and co‐workers modified the surface of ZnO with 3‐aminopropanioc acid (C3‐SAM) to achieve the optimal morphology of perovskite film.[[qv: 12c]] We introduced [6,6]‐Phenyl‐C61‐butyric Acid Methyl Ester (PCBM) thin layer to the interface between the SnO_2_ and perovskite, which can be beneficial for the electron transporting of the device.^13^ Guo et al. added the C60 interlayer between the perovskite and ZnO with inverted structure to prevent sputtering damages on the CH_3_NH_3_PbI_3_ perovskite layer.^14^ Guo et al. incorporated the wide bandgap bathocuproine (BCP) thin film as a hole‐blocking layer between PCBM and Al to block holes in PSCs with an inverted structure.^15^ However, most of them focus on the interface modification between the ETL and the perovskite of the PSCs and little work has been devoted to the interface between SnO_2_ ETLs and electrode.^16^ The recombination mechanism at the interface of transparent conductive anode and SnO_2_ ETL needs further investigation. Magnesium oxide (MgO) is a promising wide bandgap semiconductor and a good tunneling and spintronics material, which could modify the interface and retard the electron/hole recombination.^17^ Miyasaka and co‐workers inserted a thin MgO layer at the interface between TiO_2_ mesoporous layer and TiO_2_ compact layer and replaced the TiO_2_ compact layer with MgO layer in mesoporous structure PSCs to overcome “trap state recombination within TiO_2_ compact layer.” However, they did not point out the role of MgO for carrier transport regulation and the best power conversion efficiency (PCE) of device is only 11.8%.^10^ Incorporating an MgO extra nanolayer as hole‐blocking interfacial layer to the interface between the electrode and SnO_2_ ETL can be a viable way to improve the interfacial behavior and enhance the performance of the PSCs based on SnO_2_ ETL. However, to the best of our knowledge, studies on controlling the interface between SnO_2_ ETLs and transparent conductive electrode for PSCs were seldom reported.

In this work, we deposited ultrathin MgO layer on anode surface by using a sol‐gel method to form an MgO/SnO_2_ bilayer, realizing efficient electron transporting and hole‐blocking properties. The MgO layer was found to significantly avoid recombination of electrons and holes, and reduce energy loss at the interface. By employing an MgO nanolayer on FTO, we observed that the PCE was increased from 16.43% to 18.23%. When FTO was replaced with ITO, a higher power conversion efficiency of 18.82% was demonstrated. Our results reveal recombination loss mechanism at anode/SnO_2_ interface with a n‐i‐p junction working mechanism and present a new direction to improve the performance of the PSCs based on SnO_2_ ETL by electrode surface modification using MgO as hole‐blocking layer.

## 2 Results and Discussion

**Figure 1:**
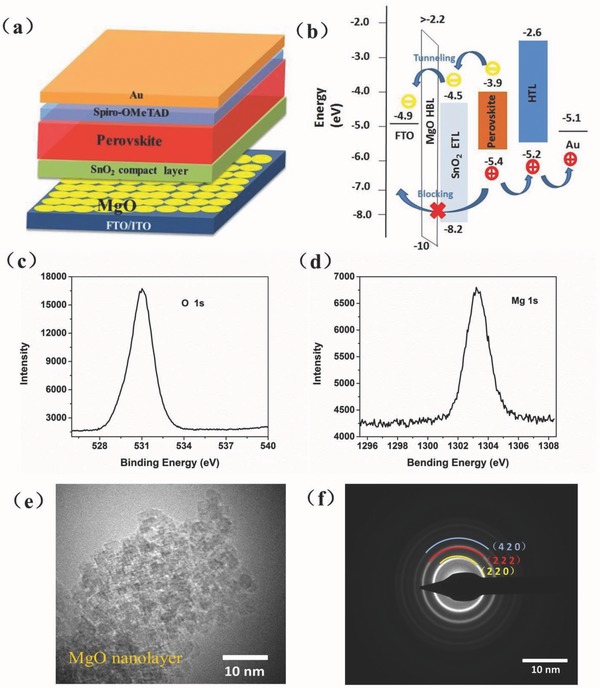
a) Schematic view of the device structure and b) energy band diagram of the device. XPS spectra of c) Mg 1s and d) O 1s peaks for an MgO film coated on a glass substrate. e) TEM and f) SAED images of an MgO nanocrystalline film.

**Figure** **1**a presents the scheme of PSCs with regular structure in this study: an FTO or ITO‐coated glass as the anode, an MgO nanolayer as the HBL (MgO was discontinuously distributed on the surface of the anode), an SnO_2_ thin film as the ETL, a perovskite absorber layer (CH_3_NH_3_PbI_3_), a 2,2,7,7‐tetrakis‐(*N*,*N*‐di‐*p*‐methoxyphenylamine)‐9,9‐spirobifluorene (spiro‐OMeTAD) as the hole transport layer (HTL), and Au as the back electrode. The energy band diagram is shown in Figure 1b. As MgO has a large energy bandgap of ≈7.8 eV,[[qv: 17b]] it ensured efficient blocking charge recombination in the process of charge transport from perovskite absorber layer to FTO negative electrode. The lower valence band position can enhance the ability of blocking the holes, and the electrons can easily tunnel through the MgO film because the MgO is a good tunneling material and the film is very thin.^20^

The MgO HBL was deposited on the FTO substrates by spin‐coating process. In order to confirm the presence of an MgO nanolayer on the FTO, X‐ray photoelectron spectroscopy (XPS) was performed. The main binding energy of 1303.21 eV shown in Figure 1c is attributed to the Mg 1s peak, and the binding energies of 531.01 eV correspond to the O 1s peak, which is the O^2−^ state in MgO, as shown in Figure 1d. The presence of the Mg and O suggest that the MgO film was successfully introduced onto the FTO anode. Then, we conducted a transmission electron microscopy (TEM) study of the prepared MgO nanolayer to characterize its morphology and crystallinity. As shown in Figure 1e, an ultrathin MgO layer (≈1–2 nm thick) has been formed uniformly. The corresponding selective area electron diffraction (SAED) images were observed (Figure 1f), which implied the polycrystalline structure of the MgO nanolayer. This result supports that the MgO nanolayer was coated onto the FTO anode.

**Figure 2:**
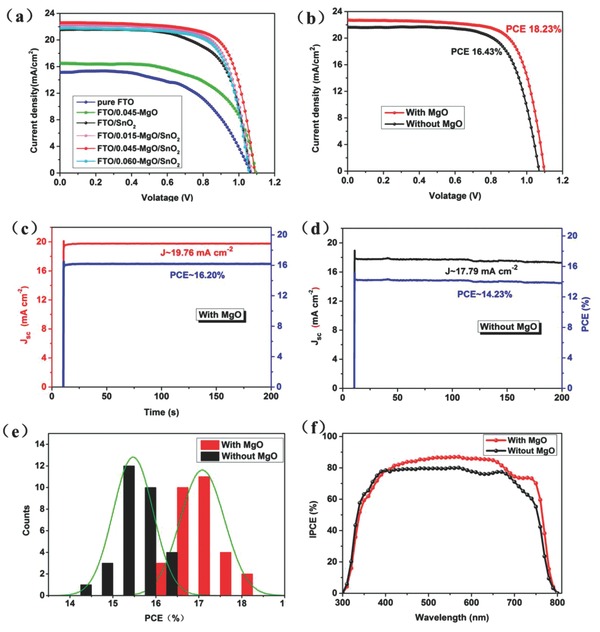
a) *J*–*V* curves of the PSCs without and with different MgO films based on FTO anode. b) The best performance of the PSCs with 0.045‐MgO HBL and without MgO HBL. Steady‐state efficiencies of the PSCs. Steady‐state efficiency of the SnO_2_‐based PSCs c) with and d) without an MgO HBL at constant bias voltages of 0.82 and 0.80 V, respectively. e) A histogram of PCEs for 30 cells of the PSCs with and without MgO HBL measured under reverse voltage scanning. f) IPCE spectra of the PSCs with and without MgO HBL.

To understand the effect after introducing an MgO nanolayer, we first optimized the thickness of the MgO film by changing the concentration of the precursor solution. Different precursor concentrations (0.015, 0.045, and 0.060 m) were obtained by adding different amount of magnesium acetate into a fixed volume of deionized (DI) water. Here, the resultant MgO films were named after 0.015‐MgO, 0.045‐MgO, and 0.060‐MgO, respectively. The PSCs based on different concentrations MgO HBL were fabricated and the photovoltaic performances of these devices were measured under 100 mW cm^−2^ (AM 1.5 simulated irradiation) illumination with a reverse scan rate of 0.1 V s^−1^. **Figure** **2**a shows the *J–V* curves of the PSCs varied with the concentration of the MgO and the detailed photovoltaic parameters are summarized in Table S1 (Supporting Information). To make a parallel comparison, except for the concentration of MgO, the devices were fabricated under the same conditions. It is clear that the PCEs of the PSCs increased first and then decreased with increasing concentration of MgO HBL. For the 0.015‐MgO thin film, the impact of MgO insertion layer on the PSC is not obvious, it may be too thin to form a continuous and compact layer, and might not be thick enough to passivate the FTO surface defects and prevent charge recombination effectively. However, the MgO films obtained from a high concentration precursor solution, such as 0.060 m, are too thick to act as an efficient HBL. Those thick films will restrict the electron injection from the perovskite absorber layer to FTO cathode, which is partially responsible for the lower *J*_sc_ and *V*_oc_. So the MgO film with a suitable thickness can suppress the charge recombination effectively and enable electrons to tunnel through the MgO HBL as well. Finally, we found that the 0.045 m is the optimal molarity of precursor to produce high‐efficiency PSCs. Besides, we also fabricated the PSCs without SnO_2_ ETL. Surprisingly, the PSCs got better performance when MgO nanolayer was incorporated between perovskite and FTO.

The best performance of the PSCs with and without MgO HBL is shown in Figure 2b, and the detailed photovoltaic parameters are summarized in **Table** **1**. The PSC with 0.045‐MgO HBL achieved a champion PCE of 18.23%, an open‐circuit voltage (*V*_oc_) of 1.10 V, a short circuit current density (*J*_sc_) of 22.7 mA cm^−2^, and a FF of 0.73. In contrast, the PSC without MgO HBL has a lower PCE of 16.43%, with a *V_oc_* of 1.07 V, a *J_sc_* of 21.63 mA cm^−2^, and a FF of 0.71. The corresponding steady‐state efficiencies are also measured and the results are shown in Figure 2c,d. The PSCs with MgO HBL achieved a steady‐state current density of 19.76 mA cm^−2^ and a steady‐state efficiency of 16.20% at a constant bias voltage of 0.82 V. Whereas the PSCs without MgO HBL achieved a lower steady‐state current density of 17.79 mA cm^−2^ and a steady‐state efficiency of 14.23% at a constant bias voltage of 0.80 V.

**Table 1:** Photovoltaic parameters for the best performance PSCs without and with different MgO films

	*V*_OC_ [V]	*J*_SC_ [mA cm^−2^]	FF	PCE [%]
Without MgO	1.07	21.63	0.71	16.43
With MgO	1.10	22.70	0.73	18.23

To check the reproducibility of the devices, we fabricated 30 cells without and with 0.045‐MgO HBL. The corresponding histogram of PCE of PSCs is shown in Figure 2e and the average device performance parameters including *V*_oc_, *J*_sc_, FF, and PCE are summarized in Table S2 (Supporting Information). The performance of each device varies little, which indicates the devices have good reproducibility. It is conspicuous that all the photovoltaic parameters of devices have notably improved after introducing an MgO nanolayer. These results proved the positive effect of MgO HBL on PSCs performance enhancement.

For simplicity, we choose two representative PSCs based on FTO anode with 0.045 m MgO HBL and without MgO HBL to compare other aspects of the performance in the following. As shown in Figure 2f, the incident photon‐to‐current conversion efficiencies (IPCEs) were measured to verify the trend of *J*_sc_ in the *J*–*V* curve for two kinds of devices with and without MgO HBL. The device with the MgO HBL demonstrates a higher IPCE, especially in the range of 400–700 nm, and the maximum IPCE of the devices with MgO HBL exceeded 90%. This higher IPCE benefits from employment of an MgO HBL, which can be associated with retarding charge recombination at the interface and reducing leakage current.

**Figure 3:**
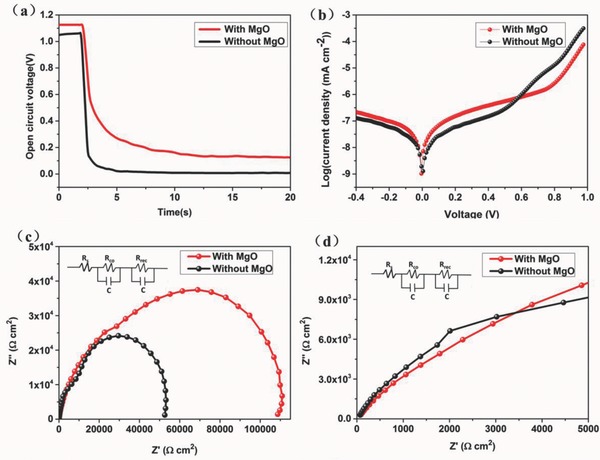
a) The OCVD curves of the perovskite solar cells with and without MgO HBL. b) *J*–*V* characteristics of devices plotted on a semilog scale and measured in the dark. Nyquist plots of the perovskite solar cells with and without ESLs, c) complete range, and d) zoom at high‐frequency range. Inset: the equivalent circuit for the cells.

To further explore the effect of the MgO on improvement of device performance, the open‐circuit photovoltage decay (OCVD), dark *J–V* characteristics, and electrochemical impedance spectroscopy (EIS) were measured. In order to elucidate the effect of MgO HBL on the charge transport, we measured the OCVD to illustrate the electron lifetime.^21^ **Figure** **3**a illustrates the voltage decay curves of the perovskite solar cells with or without MgO HBL. In this measurement, the decay of photovoltage was recorded under dark condition. We can get the information about the electron–hole recombination process from the high voltage region and the exponential increase region. As a result, the PSCs with MgO HBL exhibited a higher *V*_oc_ and longer *V*_oc_ decay time than the cell without MgO HBL. This result indicates that the cell using MgO HBL has a much longer carrier lifetime and lower interface recombination rate than the cell without MgO HBL, which is beneficial for increasing the FF and *V*_oc_.^22^

Figure 3b shows that the PSCs with MgO HBL possess smaller leakage current than PSCs without MgO HBL. The lower dark current indicates the MgO HBL could prevent the current leakage, which is beneficial for the improvement of *J*_sc_ and FF. The corresponding *J–V* curves of the PSCs with and without MgO HBL measured in the dark was shown in Figure S1 (Supporting Information). The *V*_oc_ has been evaluated according to the intercept of the linear portion of the curve to the voltage axis. The results indicate that the PSCs with MgO HBL exhibit higher *V*_oc_, which is in agreement with the result of Figure 2b.

EIS measurements were also carried out to investigate the interface charge transport and recombination in perovskite solar cells.[[qv: 7a]] Figure 3c shows the Nyquist plots of the PSCs with and without MgO HBL in the dark condition. The recombination resistance (*R*_rec_) of the PSCs can be calculated from the radius of the semicircle in the low‐frequency range. The PSCs with MgO HBL have a larger semicircle diameter and a bigger *R*_rec_, indicating the MgO HBL could suppress the electron recombination at the perovskite/FTO interfaces. Consequently, the bigger *R*_rec_ is in good agreement with higher FF of the PSCs. Figure 3d shows the incomplete semicircle in the high‐frequency range which is ascribed to contact resistance (*R*_co_) and capacitance. The PSCs with MgO HBL exhibited lower *R*_co_ value, suggesting that the charge extraction is more efficient at the ETL/perovskite interface. The slightly lower *R*_co_ can be mainly attributed to the fact that the ETL deposited on the MgO‐modified FTO exhibits more flat and uniform surface. The lower *R*_co_ values can contribute to the high *J*_sc_ value of PSCs. The results of EIS are consistent with our *J*–*V* results in Figure 2. In addition, the value of the intercept on the real axis at high frequency corresponds to the series resistance *R*_s_. it is noted that the *R*_s_ of PSCs with and without MgO are almost equal, suggesting that the thin MgO film did not influence the *R*_s_ of the PSCs obviously.

To further understand why the PSCs with MgO exhibit better device performance, the series resistance (*R*_s_), shunt resistance (*R*_sh_), and ideality factor (*A*) of the PSCs with and without MgO HBL were calculated by the diode equation.^23^ The detailed parameters are summarized in Table S3 (Supporting Information). The plots of −d*V*/d*J* versus (*J*_sc_ − 1)^−1^ and linear fitting curves of the PSCs based on the solar cells with and without MgO HBL under illumination are shown in Figure S2 (Supporting Information). The series resistance (*R*_s_) and ideality factor (*A*) can be calculated from the intercept and slope of the linear fitting results.^18^ As a result, the MgO HBL did not influence the *R*_s_ of the PSCs obviously because the MgO layer was very thin and the electrons can easily tunnel through the MgO layer. In a PSC, the quality of a junction and the carrier recombination mechanism can be represented by the values of the ideality factor.^23^ As can be seen in Figure S2 (Supporting Information), the PSCs with MgO HBL have a lower value of *A*, which indicates that the MgO HBL contributes to the quality of the junction and influence the carrier recombination. As shown in Table S3 (Supporting Information), the PSCs with MgO HBL have larger shunt resistance (*R*_sh_), which was determined from the inverse of the slope of *J–V* curves at short‐circuit current point. The better quality of the junction and the enhancement of the *R*_sh_ can decrease the current leakage, which is consistent with Figure 3b. A thin MgO HBL can sufficiently block holes and thus the photovoltaic parameters were significantly improved.

**Figure 4:**
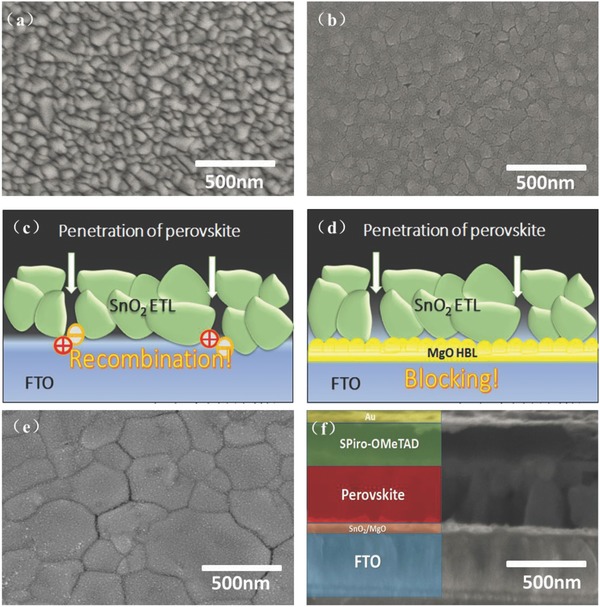
a) Top‐view SEM images of FTO glass substrate and SnO_2_ film deposited on the pure FTO. b) An illustration depicting the MgO HBL can effectively block the penetration of perovskite on the FTO surface. c) The perovskite can directly contact the FTO surface along a shunt pathway in the absence of SnO_2_ ETLs. d) The MgO HBL can inhibit the penetration of perovskite reaching the FTO surface. e) SEM image of a perovskite absorber layer surface. f) Cross‐section SEM image of a device.

The surface morphologies of the FTO, SnO_2_ ETL, and perovskite films have a significant impact on the photovoltaic performance. Herein, both scanning electron microscope (SEM) and atomic force microscopy (AFM) were employed to investigate how the additional MgO HBL influences the surface morphology and charge transportation. **Figure** **4**a shows the SEM images of the pristine FTO; we found that the surface of the FTO is very rough, which may cause rough SnO_2_ film formation and therefore the severe recombination. For the SnO_2_ ETL deposition, the surface morphology of the substrate has detrimental effects on the formation of flat and dense SnO_2_ film.

As we know, the uniform, dense, and pinhole‐free ETL is a critical factor for PSCs performance. Surface defects can introduce the recombination center level into the gap of semiconductor, which can affect the charge transport. From Figure 4b, we found that the ETL prepared by sol‐gel method was unable to fully cover the surface of the FTO electrode and exhibited some pinholes and cracks. AFM was employed to further elucidate how the MgO layer influences the surface morphology of FTO substrate and ETLs (Figure S3, Supporting Information). AFM was employed under normal atmospheric air conditions and without any treatment on FTO substrate and the surface of the MgO. The AFM images confirmed that the FTO substrate with MgO Film was smoother than the pure FTO substrate film. The root‐mean‐square (RMS) roughness of FTO substrates with MgO Film and pure FTO substrate are 12.4 and 18.1 nm, respectively. The lower roughness implied the MgO film passivated the FTO surface defects, filled the gaps between FTO crystals, and made the surface more uniform, which is very helpful to reduce the recombination at the interface. Besides, we noticed that the ETLs deposited on the MgO‐modified FTO substrate possessed a slightly decreased RMS compared with the ETLs directly spin‐casted on pure FTO. The RMS are 5.17 and 8.01 nm, respectively. As a result, ETLs with improved smoothness and coverage were obtained after MgO modification. The result suggests that the morphology of the ETLs in planar PSCs is determined by the surface roughness of the FTO. Reduced recombination can be realized by decreasing the shunting pathways via a flat and uniform ETL, which also corresponds to a higher photovoltaic parameters. The rough ETLs would generate a rough and inhomogeneous surface, creating the surface defects.

As illustrated in Figure 4c, the perovskite spin‐coated on the ETL may penetrate through the pinholes between the SnO_2_ grains. So, this may cause a direct contact between FTO substrate and perovskite absorber, which can trigger severe current leakage and the electron–hole recombination at the interface. To impede the detrimental contact between the FTO and perovskite, we incorporated a wide bandgap dielectric MgO interlayer to form a bilayer structure with SnO_2_. As we can see from Figure 4d, the MgO HBL can inhibit the penetration of perovskite to the FTO surface. Thereby, it can minimize the possibility of current leakage and electron–hole recombination and improve the performance of the device. Figure 4e presents the morphology of CH_3_NH_3_PbI_3_ layer. This morphology of the perovskite is consistent with the perovskite prepared by antisolvent technique.^24^ Figure 4f presents the cross‐section SEM image of a completed device, which consists of an FTO glass substrate, SnO_2_/MgO bilayer with a thickness of 50 nm, perovskite layer with a thickness of 500 nm, spiro‐MeOTAD with a thickness of 300 nm, and Au counter electrode with a thickness of 60 nm.

**Figure 5:**
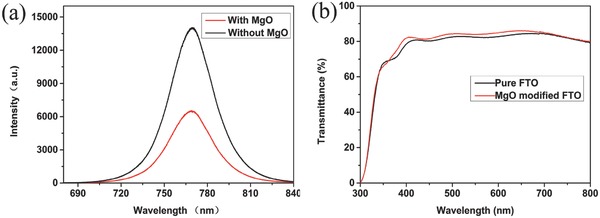
a) Steady‐state PL spectra of CH_3_NH_3_PbI_3_ contacted with ETL and ETL/MgO HBL. b) Transmittance spectra of pristine FTO and MgO modified FTO.

It is expected that the electrons generated in perovskite layer can be transported and collected more efficiently. Steady‐state photoluminescence (PL) characterizations were carried out to further investigate the charge transporting properties and recombination of charges of the corresponding devices. Steady‐state PL spectra of ETLs and ETL/MgO are shown in **Figure** **5**a. Perovskite film exhibits an emissive band peaked at around 770 nm. The perovskite film deposited on the ETL/MgO/FTO substrate exhibited a significant fluorescence quenching behavior when compared with that on ETL/FTO. The much lower PL intensity indicates that the ETL/MgO bilayer plays an important role in facilitating the charge transfer and hindering the recombination between electrons and holes. The improvement of the charge transporting may contribute to the change of the SnO_2_ surface morphology and the pivotal role of MgO HBL in hindering the recombination. This fact is consistent with the contact resistance values analyzed in EIS.

It is notable that the MgO thin films coated on FTO glass substrates are antireflective (Figure 5b). The MgO‐modified FTO improve the light transmittance slightly compared to the pristine FTO. The better optical property facilitates the generation of the electron–hole pairs, which improves the *J*_sc_. The better optical transmission properties can be attributed to the smoother surface when FTO is covered with MgO thin film. The transmission spectra of FTO/SnO_2_ and FTO/MgO/SnO_2_ are presented in Figure S4 (Supporting Information). We also observed better transmittance when MgO was incorporated into the interface between the FTO and SnO_2_.

To further explore the effect of the MgO HBL on PSCs with different anodes, we replaced the FTO anode with ITO anode. As shown in Figure S5 (Supporting Information), for the PSCs with regular structure (ITO/SnO_2_/CH_3_NH_3_PbI_3_/spiro‐OMeTAD/Au), the device achieved a PCE of 16.92% and a steady‐state efficiency of 13.66%. When an MgO nanolayer is incorporated between ITO and SnO_2_ ETL, the PSC exhibited better performance with a PCE of 18.82% and a steady‐state efficiency of 17.85%. For the PSCs without SnO_2_ ETL, the PSCs also got better performance when MgO nanolayer is incorporated between perovskite and ITO. The detailed photovoltaic parameters are summarized in Table S4 (Supporting Information). The results indicate that the MgO nanolayer can enhance the performance of the PSCs with different transparent conductive anode.

## 3 Conclusion

By utilizing an ultrathin MgO insulating nanolayer at the interface of anode and SnO_2_ ETL, improved device performance for planar perovskite solar cells was demonstrated. The enhancement mechanism was studied systematically by using AFM, PL, EIS, OCVD measurements, and diode model calculations. It was found that thin layer of MgO can make the FTO smoother and enhance the transmission of FTO. Most importantly, MgO layer can facilitate the hole blocking and suppress the electron–hole recombination at the FTO/SnO_2_ ETL interface owing to its wide bandgap and insulating properties. Finally, devices with MgO HBL layer yield a PCE of over 18%, which was greatly improved than device without MgO HBL. Our work points out the role of MgO in charge carrier transport regulation and reveals the effect of recombination at anode/SnO_2_ interface with a n‐i‐p junction working mechanism. Besides, we demonstrate the universal application of MgO on different anodes and provide a novel interface engineering strategy on the road to improving the performance of the PSCs based on SnO_2_ ETL.

## 4 Experimental Section

*Materials*: FTO glass with a sheet resistance of 14 Ω sq^−1^ and ITO glass with a sheet resistance of 10 Ω sq^−1^ were purchased from Asahi Glass (Japan). Magnesium acetate (Aladdin, 99.99%) was dissolved in deionized water to prepare aqueous magnesium acetate. SnCl_2_·2H_2_O (Alfa, 99.9985%) was used to prepare precursor solutions. Methylammonium iodide (MAI) was prepared according to a literature.^18^ Lead iodide (PbI_2_) was purchased from Aladdin reagent. MAI and PbI_2_ (1:1, mol mol^−1^) were dissolved in the mixed solution of dimethyl sulfoxide (DMSO) and *N*,*N*‐dimethylformamide (DMF) with a concentration of 1.38 m. The solution was stirred at 60 °C for 12 h inside an argon glovebox. The hole transport material was made up of 68 × 10^−3^ m spiro‐OMeTAD (Shenzhen Feiming Science and Technology Co., Ltd., 99.0%), 55 × 10^−3^ m TBP (Aladdin reagent), and 26 mg Li‐TFSI (Aladdin reagent) in acetonitrile and chlorobenzene (1:10 in volume ratio). The purity of gold wire is 99.99%.

*Solar Cell Fabrication*: FTO and ITO glass substrates were sequentially rinsed by sonication in detergent, DI water, acetone, and ethanol, and finally dried in bake oven. After that, the FTO and ITO glasses were modified with MgO by spin‐coating precursor solutions with different concentrations at 4000 rpm for 30 s according to a previous literature.[[qv: 17b]] SnO_2_ thin films were spin‐coated on the FTO and ITO substrates or MgO‐modified FTO and ITO substrates by a modified sol‐gel method similar to ref. [[qv: 6a]]. The spinning rate was set at 2000 rpm for 45 s. Then the SnO_2_ thin films underwent gradient annealing and finally heated in air at 200 °C for 1 h. Perovskite (MAPbI_3_) absorber layers were deposited in the samples by one‐step method.^19^ First, MAPbI_3_ precursor solution was spread onto substrates. Then, the spin–coater was started at a rotation speed of 1000 rpm for 5 s and 4000 rpm for another 40 s. Chlorobenzene was drop‐casted quickly after the 4000 rpm spin‐coating started. The perovskite films were then heated at 100 °C on a hotplate for 10 min. The spiro‐OMeTAD solution was spin‐coated on perovskite film at 3000 rpm for 30 s. Finally, a thin gold electrode was deposited by thermal evaporation.

*Characterization*: The morphologies of FTO, SnO_2_, and CH_3_NH_3_PbI_3_ film observation were conducted by a high‐resolution field emission SEM (JSM 6700F, Japan). The morphology and crystallinity of MgO film was observed by a JEOL‐2010 TEM. The morphologies and roughness of the surfaces of bare FTO and MgO‐coated FTO substrates were characterized by AFM (SPM‐9500j3). Compositions of the MgO films were measured by an XPS system (Thermo Scientific, Escalate 250Xi). *J*–*V* characteristics of solar cells were characterized on a CHI 660D electrochemical work station (Shanghai Chenhua Instruments, China) with a standard ABET Sun 2000 Solar Simulator. All the cells were performed under a 100 mW cm^−2^ (AM 1.5 simulated irradiation) illumination and the scan rate was set as 0.1 V s^−1^. The area of the Au electrode was 0.09 cm^2^. The transmission spectra of the MgO films coated on transparent conductive oxide substrates or pure transparent conductive oxide were examined by an ultraviolet–visible spectrophotometer (CARY5000, Varian) within a wavelength range of 300–800 nm. IPCE was conducted by a QE/IPCE system (Enli Technology Co. Ltd.) in the wavelength range from 320 to 800 nm. Steady‐state PL was obtained with a 532 nm laser, as the excitation source, pulsed at a frequency of 9.743 MHz.

## Conflict of Interest

The authors declare no conflict of interest.

## Supporting information

Supplementary

## References

[1] a) B. Wang , T. Chen , Adv. Sci. 2016, 3, 1500262; 10.1002/advs.201500262 PMC5054937 27774390

[2] a) C. Zuo , L. Ding , Nanoscale 2014, 6, 9935; 25074642 10.1039/c4nr02425g

[3] a) S. Kazim , M. K. Nazeeruddin , M. Gratzel , S. Ahmad , Angew. Chem. 2014, 53, 2812; 24519832 10.1002/anie.201308719

[4] Y. Li , L. Meng , Y. M. Yang , G. Xu , Z. Hong , Q. Chen , J. You , G. Li , Y. Yang , Y. Li , Nat. Commun. 2016, 7, 10214. 26750664 10.1038/ncomms10214 PMC4729901

[5] a) J. You , L. Meng , T. B. Song , T. F. Guo , Y. M. Yang , W. H. Chang , Z. Hong , H. Chen , H. Zhou , Q. Chen , Y. Liu , N. De Marco , Y. Yang , Nat. Nanotechnol. 2016, 11, 75; 26457966 10.1038/nnano.2015.230

[6] a) W. Ke , G. Fang , Q. Liu , L. Xiong , P. Qin , H. Tao , J. Wang , H. Lei , B. Li , J. Wan , G. Yang , Y. Yan , J. Am. Chem. Soc. 2015, 137, 6730; 25987132 10.1021/jacs.5b01994

[7] a) T. Meng , C. Liu , K. Wang , T. He , Y. Zhu , A. Al‐Enizi , A. Elzatahry , X. Gong , ACS Appl. Mater. Interfaces 2016, 8, 1876; 26727027 10.1021/acsami.5b09873

[8] W. Ke , D. Zhao , A. J. Cimaroli , C. R. Grice , P. Qin , Q. Liu , L. Xiong , Y. Yan , G. Fang , J. Mater. Chem. A 2015, 3, 24163.

[9] a) V.‐D. Dao , L. L. Larina , H.‐S. Choi , Thin Solid Films 2015, 593, 10;

[10] A. Kulkarni , A. K. Jena , H.‐W. Chen , Y. Sanehira , M. Ikegami , T. Miyasaka , Solar Energy 2016, 136, 379.

[11] J. Shi , X. Xu , D. Li , Q. Meng , Small 2015, 11, 2472. 25688549 10.1002/smll.201403534

[12] a) Z. Zhu , C. C. Chueh , F. Lin , A. K. Jen , Adv. Sci. 2016, 3, 1600027; 10.1002/advs.201600027 PMC5039977 27711269

[13] W. Ke , C. Xiao , C. Wang , B. Saparov , H. S. Duan , D. Zhao , Z. Xiao , P. Schulz , S. P. Harvey , W. Liao , W. Meng , Y. Yu , A. J. Cimaroli , C. S. Jiang , K. Zhu , M. Al‐Jassim , G. Fang , D. B. Mitzi , Y. Yan , Adv. Mater. 2016, 28, 5214. 27145346 10.1002/adma.201600594

[14] W.‐C. Lai , K.‐W. Lin , T.‐F. Guo , P. Chen , Y.‐T. Wang , Appl. Phys. Lett. 2015, 107, 253301.

[15] J. Y. Jeng , K. C. Chen , T. Y. Chiang , P. Y. Lin , T. D. Tsai , Y. C. Chang , T. F. Guo , P. Chen , T. C. Wen , Y. J. Hsu , Adv. Mater. 2014, 26, 4107. 24687334 10.1002/adma.201306217

[16] a) Y. Liu , L. A. Renna , Z. A. Page , H. B. Thompson , P. Y. Kim , M. D. Barnes , T. Emrick , D. Venkataraman , T. P. Russell , Adv. Energy Mater. 2016, 6, 1600664;

[17] a) G. S. Han , H. S. Chung , B. J. Kim , D. H. Kim , J. W. Lee , B. S. Swain , K. Mahmood , J. S. Yoo , N.‐G. Park , J. H. Lee , H. S. Jung , J. Mater. Chem. A 2015, 3, 9160;

[18] A. Bose , A. K. Shukla , K. Konishi , S. Jain , N. Asam , S. Bhuktare , H. Singh , D. D. Lam , Y. Fujii , S. Miwa , Y. Suzuki , A. A. Tulapurkar , Appl. Phys. Lett. 2016, 109, 032406.

[19] a) J. Wang , M. Qin , H. Tao , W. Ke , Z. Chen , J. Wan , P. Qin , L. Xiong , H. Lei , H. Yu , G. Fang , Appl. Phys. Lett. 2015, 106, 121104;

[20] N. K. Elumalai , A. Uddin , Energy Environ. Sci. 2016, 9, 391.

[21] J. Shi , J. Dong , S. Lv , Y. Xu , L. Zhu , J. Xiao , X. Xu , H. Wu , D. Li , Y. Luo , Q. Meng , Appl. Phys. Lett. 2014, 104, 063901.

[22] M. Qin , J. Ma , W. Ke , P. Qin , H. Lei , H. Tao , X. Zheng , L. Xiong , Q. Liu , Z. Chen , J. Lu , G. Yang , G. Fang , ACS Appl. Mater. Interfaces 2016, 8, 8460. 26996215 10.1021/acsami.5b12849

[23] N. J. Jeon , J. H. Noh , Y. C. Kim , W. S. Yang , S. Ryu , S. I. Seok , Nat. Mater. 2014, 13, 897. 24997740 10.1038/nmat4014

[24] Y. Wu , X. Yang , W. Chen , Y. Yue , M. Cai , F. Xie , E. Bi , A. Islam , L. Han , Nat. Energy 2016, 1, 16148.

