# Supplementary material for: MgO Nanoparticle Modified Anode for Highly Efficient SnO2‐Based Planar Perovskite Solar Cells
Source: Adv Sci (Weinh). 2017 May 2;4(9):1700031. doi: 10.1002/advs.201700031 (PMC5604382; doi:10.1002/advs.201700031)
Supplement: Supplementary file 1 — Supplementary [file ADVS-4-na-s001.pdf]

Copyright WILEY-VCH Verlag GmbH & Co. KGaA, 69469 Weinheim, Germany, 2017.

Supporting Information

for *Adv. Sci.,* DOI: 10.1002/advs.201700031

MgO Nanoparticle Modified Anode for Highly Efficient SnO_2-Based Planar Perovskite Solar Cells

*Junjie Ma, Guang Yang, Minchao Qin, Xiaolu Zheng, Hongwei Lei, Cong Chen, Zhiliang Chen, Yaxiong Guo, Hongwei Han, Xingzhong Zhao, and Guojia Fang**

MgO Nanoparticle Modified Anode for Highly Efficient SnO_2 Based Planar Perovskite Solar Cells

Junjie Ma,[a] Guang Yang,[a] Minchao Qin,[a] Xiaolu Zheng,[a] Hongwei Lei,[a] Cong Chen,[a] Zhiliang Chen,[a] Yaxiong Guo,[a] Hongwei Han,[b] Xingzhong Zhao [a] and Guojia Fang [a] *

[a] Key Lab of Artificial Micro- and Nano-Structures of Ministry of Education of China, School of Physics and Technology, Wuhan University, Wuhan 430072, People's Republic of China

[b] Wuhan National Lab on Opto-electronics, Huazhong University of Science and Technology, Wuhan 430074, People's Republic of China

*Corresponding Author. E-mail: gjfang@whu.edu.cn

Table S1. Photovoltaic parameters for the PSCs without and with different MgO films.

Molarity (M)	V_{oc} (V)	J_{sc}(mA cm^{-2})	FF	PCE (%)
Pure FTO	1.06	15.11	0.56	9.12
FTO/0.045- MgO	1.09	16.51	0.61	11.03
FTO/SnO_2	1.07	21.56	0.67	15.45
FTO/0.015- MgO/SnO_2	1.08	22.23	0.71	17.04
FTO/0.045- MgO/SnO_2	1.09	22.59	0.72	17.72

FTO/0.060- MgO /SnO_2	1.06	21.74	0.72	16.59

Table S2. The average photovoltaic parameters of PSCs on FTO without and with 0.045-MgO nanolayer.

	V_{OC} (V)	J_{SC}(mA cm^{-2})	FF	PCE (%)
Without MgO	1.04±0.04	21.48±0.47	0.69±0.02	15.43±1.20
With MgO	1.07±0.03	22.25±1.03	0.72±0.01	17.08±1.24

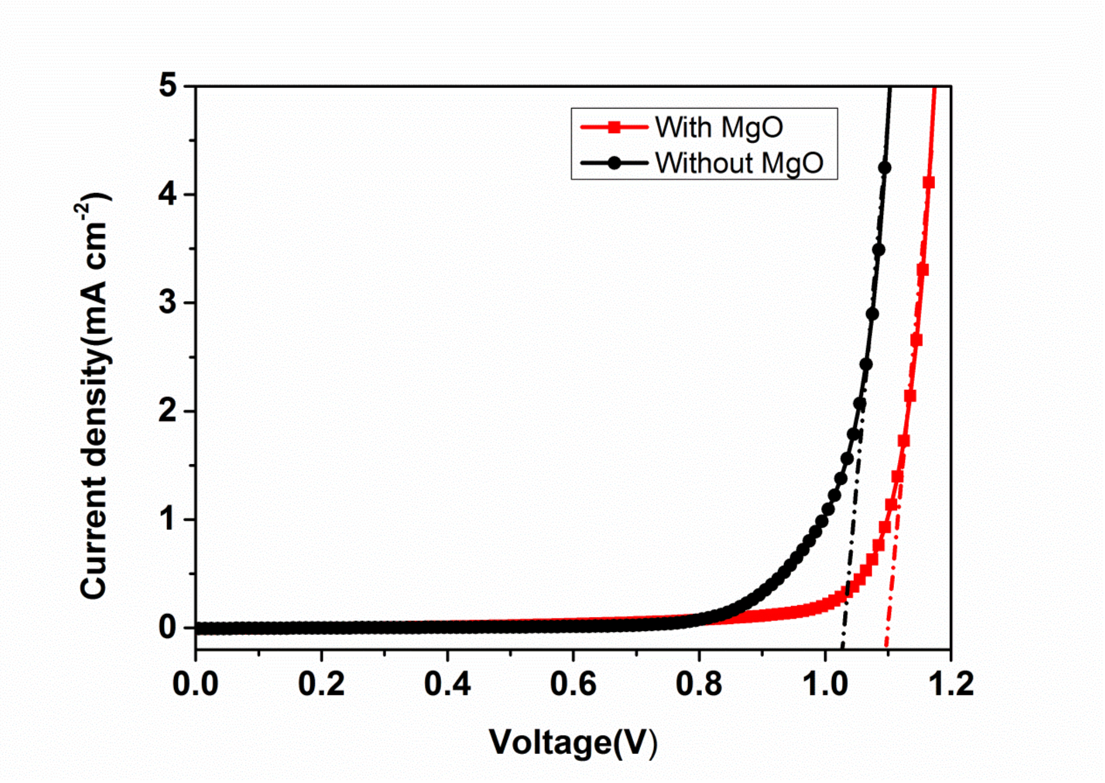

Figure S1. J-V curves of the PSCs without and without MgO films measured in the dark.

Table S3. the value of series resistance (R_s), shunt resistance(R_{sh}) and ideality factor (A) of the perovskite solar cells with or without MgO HBL.

	R_s(Ω cm^2)	R_{sh}(Ω cm^2)	A
Without MgO	2.21	2272.72	5.76
With MgO	4.85	4166.67	2.69

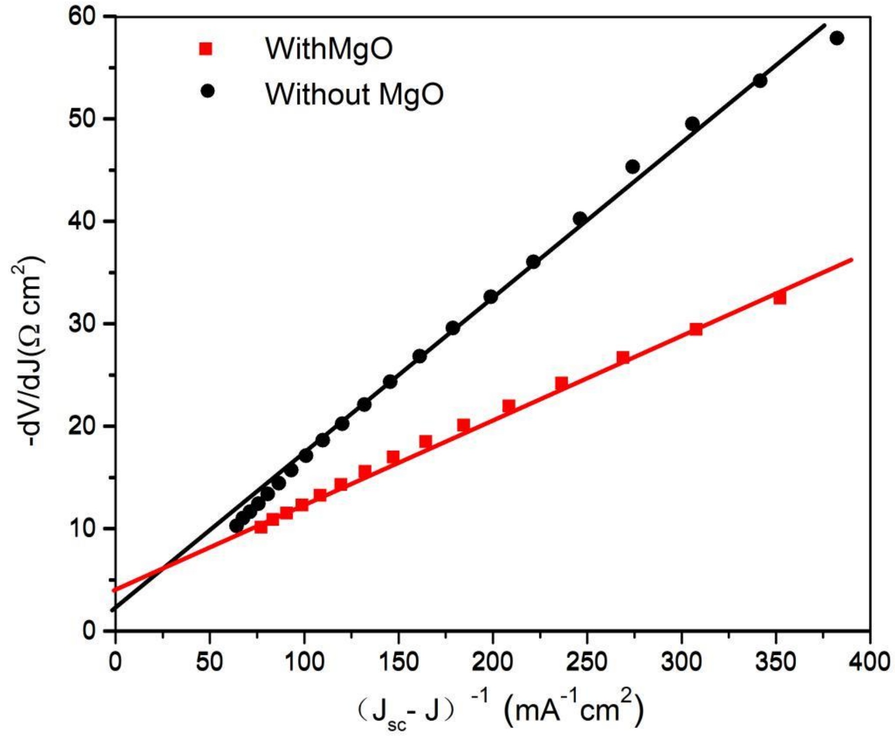

Figure S2. plots of -dV/dJ vs (JSC-1)-1 and linear fitting curves of the PSCs based on the solar cells with or without MgO HBL under illumination.

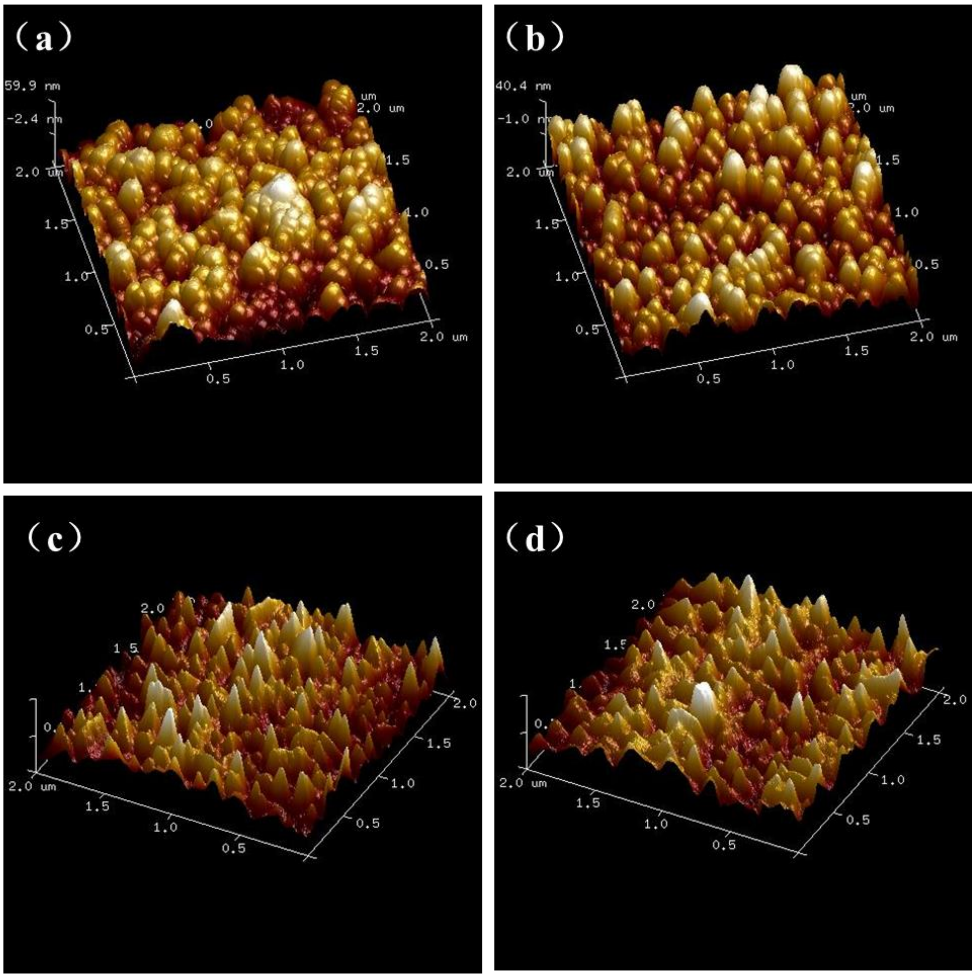

Figure S3. AFM image of a) bare FTO b) a FTO subtrate coated with MgO c) a FTO subtrate coated with SnO_2 d) FTO subtrate coated with MgO/SnO_2

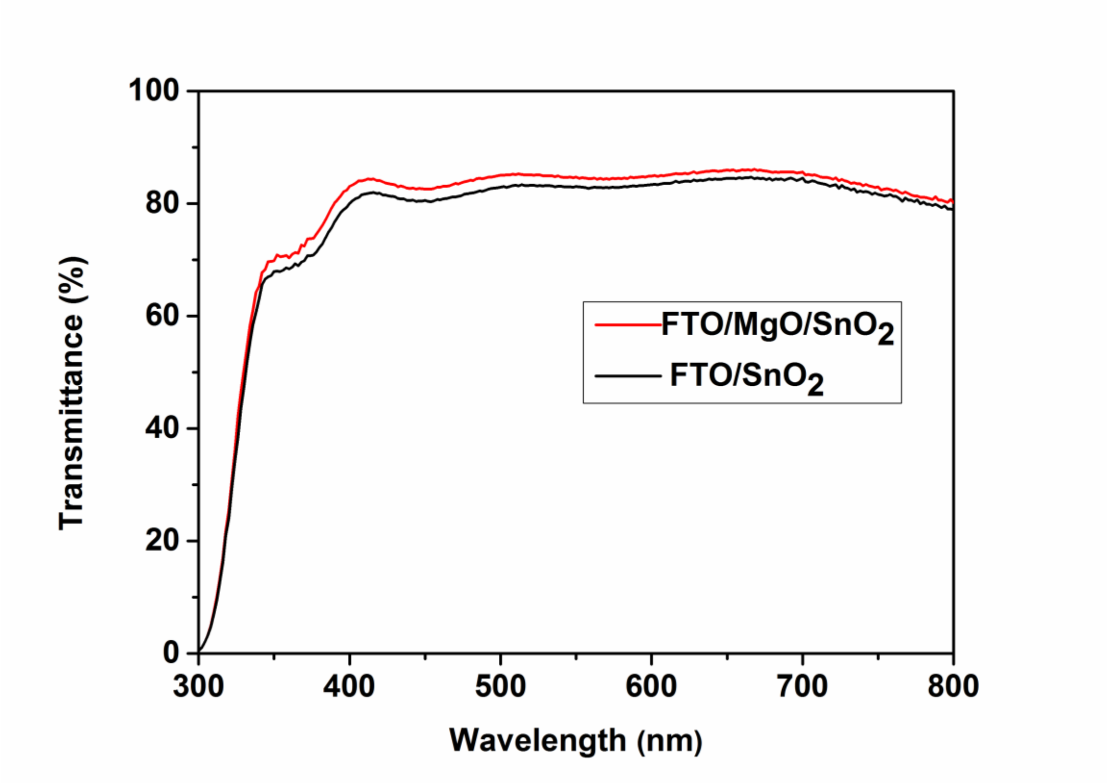

Figure S4. Transmittance spectra of FTO/SnO_2 and FTO/MgO/SnO_2.

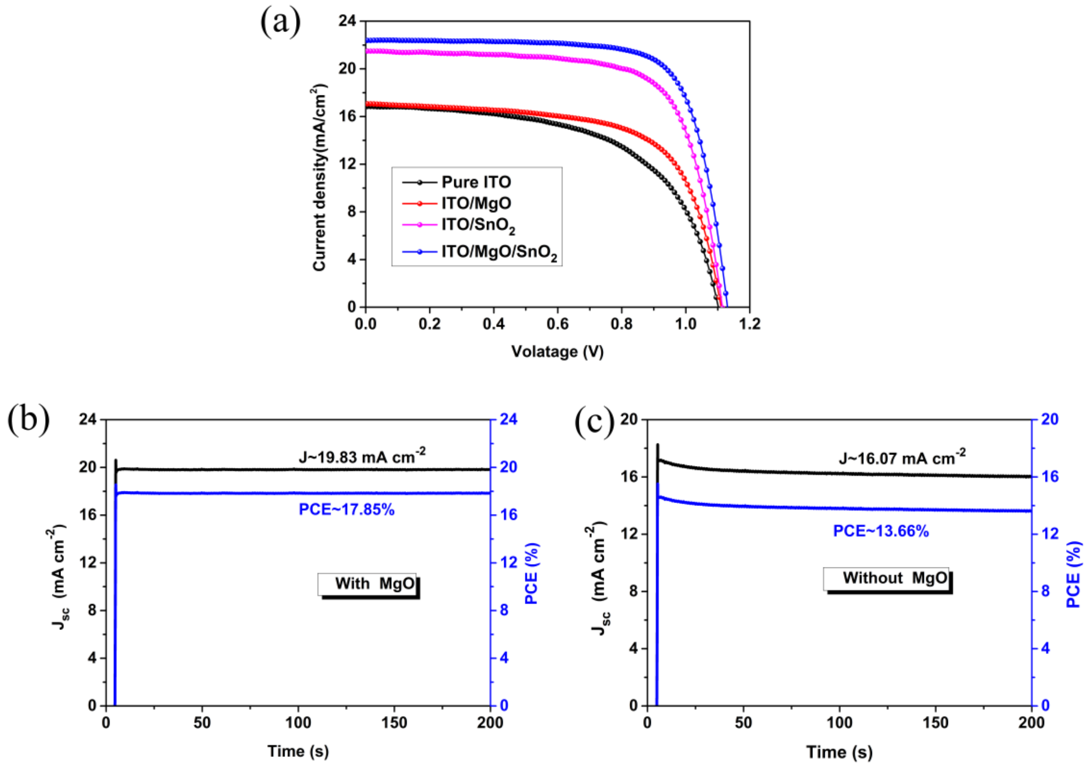

Figure S5. a) J-V curves of the PSCs without and with MgO films based on ITO anode. Steady-state efficiencies of the PSCs with SnO_2 ETL based on ITO anode. b) with and c) without a MgO HBL at a constant bias voltages of 0.90 V and 0.85 V, respectively.

Table S4. Photovoltaic parameters for the PSCs without and with MgO films based on ITO anode.

	V_{oc} (V)	J_{sc}(mA cm^{-2})	FF	PCE (%)
Pure ITO	1.10	16.84	0.58	10.79
ITO/ MgO	1.12	17.07	0.65	12.39
ITO/SnO_2	1.12	21.49	0.71	16.92
ITO/MgO/SnO_2	1.14	22.39	0.74	18.82
